# A systematic review of the usefulness of magnetic resonance imaging in predicting the gait ability of stroke patients

**DOI:** 10.1038/s41598-021-93717-4

**Published:** 2021-07-12

**Authors:** Takeshi Imura, Tsubasa Mitsutake, Yuji Iwamoto, Ryo Tanaka

**Affiliations:** 1 Department of Rehabilitation, Faculty of Health Sciences, Hiroshima Cosmopolitan University, 3-2-1, Otsuka-higashi, Hiroshima, 731-3166 Japan; 2 Department of Physical Therapy, Fukuoka International University of Health and Welfare, Fukuoka, Japan; 3 Graduate School of Humanities and Social Sciences, Hiroshima University, Hiroshima, Japan

**Keywords:** Health care, Medical research, Neurology

## Abstract

The usefulness of magnetic resonance imaging (MRI) in predicting gait ability in stroke patients remains unclear. Therefore, MRI evaluations have not yet been standardized in stroke rehabilitation. We performed a systematic review to consolidate evidence regarding the use of MRIs in predicting gait ability of stroke patients. The Medline, Cumulative Index to Nursing and Allied Health Literature, and SCOPUS databases were comprehensively searched. We included all literature published from each source’s earliest date to August 2020. We included 19 studies: 8 were classified as structure- or function-based MRI studies and 11 as neural tract integrity-based MRI studies. Most structure- or function-based MRI studies indicated that damage to motor-related areas (primary motor cortex, corona radiata, internal capsule, and basal ganglia) or insula was related to poor gait recovery. In neural tract integrity-based MRI studies, integrity of the corticospinal tract was related to gait ability. Some studies reported predictive value of the corticoreticular pathway. All included studies had some concerns, at least one, based on the Cochrane risk of bias instrument. This review suggests that MRIs are useful in predicting gait ability of stroke patients. However, we cannot make definitive conclusion regarding the predictive value, due to the lack of quantitative evaluations.

## Introduction

Gait ability is important for mobility and maintaining general health^1^. Stroke patients usually have residual disabilities; in particular, many stroke survivors experience a gait disability because of lower limb hemiparalysis, resulting in movement restrictions in daily life^2–4^. The patients with post-stroke hemiparesis frequently present with asymmetric gait patterns^5^. The asymmetric gait patterns are characterized increased or decreased swing time and stance time, (i.e., temporal asymmetry) and increased or decreased step length (i.e., spatial asymmetry)^6–8^. The altered gait pattern leads to decreased walking velocity^5^. Acquiring functional gait ability is considered a principal goal of rehabilitation, because gait affects a patient’s or family’s quality of life^9,10^. Even in cases where an individual is not expected to acquire functional gait ability, a rehabilitation program focused on substitutional locomotion, such as using a wheelchair or modifying the individual’s environment, can be meaningful for expanding an individual’s mobility.

Predicting an individual’s gait ability from the early phase after stroke onset is crucial for setting realistic rehabilitative goals and/or arranging a rehabilitation program. Previous studies reported that the initial motor and functional impairment level had predictive value for gait ability, as did specific evaluation tools, like the revised version of the Ability for Basic Movement Scale II^11,12^. Developments in the field of neuroscience have been gradually clarifying the complex regulation of the neural network for gait^13–17^, and anatomically determining the extent of damage to the gait-related neural network might have extreme value in predicting gait ability. Brain imaging, including structural imaging and functional imaging, has been widely used in clinical situations for disease diagnoses, lesion identification, or understanding recovery mechanisms^18–20^. In magnetic resonance imaging (MRI), in particular, diffusion-weighted imaging (DWI) has been used for early detection of ischemic brain lesions^18^ and diffusion tensor imaging (DTI) has been applied to describe neural tracts in recent years^21–23^. In fact, MRIs have already been used to predict a patient’s functional outcomes^24^ or ability to perform activities of daily living^25^. Stinear and Ward^26^ stated in a systematic review that imaging may help clinicians to identify each patient’s potential for recovery, set realistic rehabilitation goals, and select therapy techniques on the basis of residual connections between key elements of the central nervous system.

Skilled clinicians who empirically understand the usefulness of brain imaging have already been applying it in routine patient evaluations, including to predict gait ability. However, no systematic review has evaluated the usefulness of MRIs in predicting the gait ability of stroke patients; as a result, MRI evaluations have not been standardized in the field of stroke rehabilitation. Therefore, this systematic review aimed to consolidate evidence regarding the use of MRIs in predicting the stroke patient’s gait ability including the degree of gait independence, gait speed, or gait endurance.

## Methods

A systematic review of the literature was performed according to the Preferred Reporting Items for Systematic Review and Meta-Analysis (PRISMA) guidelines^27^. This review was registered in PROSPERO (ID: CRD 42020206355).

### Selection criteria

Studies were included in this systematic review if they met following criteria: (1) the patients were diagnosed with hemorrhagic or ischemic strokes; (2) the patients had a conventional MRI (T1-weighted imaging, T2-weighted imaging, or fluid-attenuated inversion recovery), functional MRI, DWI, or DTI; (3) gait ability outcomes were assessed; (4) the MRI was applied in predicting gait ability; (5) the study was a cohort study or case–control study; and (6) the article was written in English.

Studies were excluded if: (1) the study included patients with subarachnoid hemorrhages; (2) the study was a case study or cross-sectional study; or (3) the study was a review article.

### Search strategy and study selection

The Medline, Cumulative Index to Nursing and Allied Health Literature, and SCOPUS electronic databases were comprehensively searched. The search terms “patient”, “exposure” , and “outcome” were combined with the “AND” operator. “Patient” was defined as a stroke patient. “Exposure” was defined as an MRI evaluation. “Outcome” was defined as gait performance prediction. For each concept, we combined synonyms and Medical Subject Heading terms with the “OR” operator. There were no limits with regard to dates. The searches were performed on August 31, 2020. An example of the search strategy used in the Medline database is shown in Supplementary File 1.

The articles identified through database searching were summarized into spreadsheet that were created using Microsoft Excel 2019. After excluding duplicates, two reviewers (TI and TM) independently screened each article based on the title and abstract using predetermined eligibility criteria in order to determine relevant manuscripts for full-text review. Subsequently, full-text copies of articles that were not excluded based on the title or abstract were retrieved, and the inclusion and exclusion criteria were reapplied to these studies to determine suitability for final inclusion. Any disagreements during the article screening and selection were resolved through discussion, and decisions were made by a third person if the two reviewers could not reach a consensus.

### Data extraction

Predesigned spreadsheets that were created using Microsoft Excel 2019 were used to extract data on participants, exposures, outcome measurements, and results. Two reviewers (TI and TM) discussed and decided on the extraction data, and a third person confirmed.

### Risk of bias evaluation in individual studies

To evaluate the risk of bias in each study, two researchers (TI and TM) independently applied the tool to assess risk of bias in cohort studies (the Cochrane risk of bias instrument). The articles were evaluated using predetermined criteria (Supplementary File 2).

## Results

**Figure 1:**
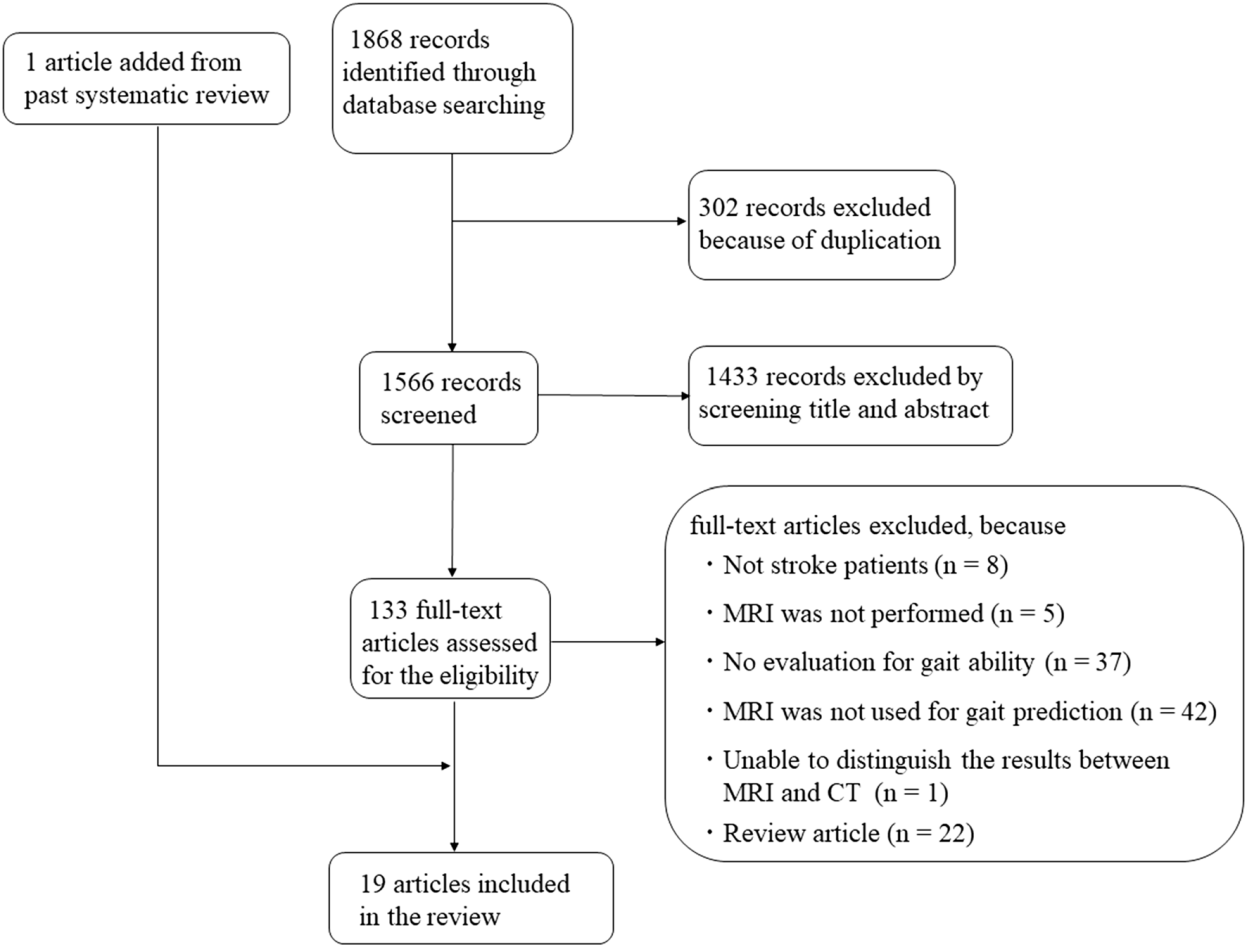
Flow diagram of included and excluded studies. *CT* computed tomography, *MRI* magnetic resonance imaging.

The combined database search identified 1868 studies (Fig. 1). After adjusting for duplicates, 1566 studies were considered. Out of these, 1433 studies did not meet the selection criteria after a review of the titles and abstracts. The complete texts of the remaining 133 studies were examined in detail, and 115 studies did not meet the inclusion criteria. One article was added from a past systematic review. Finally, 19 studies fulfilled the inclusion criteria and were included in the analysis. Critical information regarding the included studies is summarized in Table 1, including data on the study population, type of MRI evaluation, key analysis, and predictive outcomes. The average age of participants in all the studies ranged from 52.1 to 71.5 years. In addition, the stroke phase at baseline ranged from within 3 days to an average of 212 days after onset. The included studies were divided broadly into two research categories: (1) structure- or function-based MRI studies, which focused on affected brain structures or imaging findings; and (2) neural tract integrity-based MRI studies, which focused on neural tract integrity using DTI methodology.

**Table 1:** Summary of included studies.

Study: author, journal, year (retrospective or prospective)	Participants								MRI evaluation			Analysis	**Predictive outcome**	
	Size (n)	Age, years	Sex, M/F, n	Stroke type, n	Stroke location	Lesion side, R/L, n	Stroke phase at baseline	Function at baseline	Tesla	Contents	Evaluation days from onset		Outcome scale	Evaluation days from onset
Lee et al., Brain Behav, 2017^29^ (retrospective)	30	55.0 ± 13.7	17/13	Ischemic: 10 Hemorrhagic: 20	Supratentorial	15/15	Acute (within 14 days after onset)	FMA-UE: 20.1 ± 18.5 FMA-LE: 14.0 ± 8.1 FMA-S: 10.5 ± 8.4	3	T1WI T2WI	Within 14 days after onset	Overlay of lesions Subtraction analysis Voxel-based lesion symptom mapping	FAC	Initial assessment (within 14 days), 1, 3, and 6 months after onset
Kim et al., Neuroreport, 2018^39^ (not mentioned)	48	Group A: 64.0 ± 12.7 Group B: 63.9 ± 12.4 Group C: 67.9 ± 12.3	34/14	Ischemic: 40 Hemorrhagic: 8	Supratentorial	29/19	Acute to subacute (within 6 weeks after onset)	FMA (Group A: 45.4 ± 20.4, Group B: 32.4 ± 19.9, Group C: 17.2 ± 13.8)	3	DTI	Within 6 weeks after onset	3 groups comparison	FAC	Baseline (within 1 week after DTI) and at 2 years after onset
Jones et al., Hum Brain Mapp, 2016^31^ (prospective)	50	64.6 ± 15.0	28/22	Ischemic: 41 Hemorrhagic: 9	Supratentorial and infratentorial	25/25	Acute to subacute (median 16 days, range: 3–42 days)	–	1.5	T1WI T2WI FLAIR	Median 52 days after onset (range 17–74 days)	Overlay of lesions Multiple regression analysis	FAC Gait speed MRMI	At entry into the study and the end of 6 weeks of intervention phase
Yeo et al., J Stroke Cerebrovasc Dis, 2020^41^ (retrospective)	9	59.3 ± 12.4	7/2	Ischemic: 0 Hemorrhagic: 9	Infratentorial	–	Acute to subacute (15.3 ± 6.6 days)	–	1.5	T2WI DTI	15.3 ± 6.6 days after onset and 41.2 ± 21.6 days after onset	2 groups comparison	FAC	15.3 ± 6.6 days after onset and 41.2 ± 21.6 days after onset
Miyai et al., Stroke, 2000^28^ (not mentioned)	94	IC and Pt: 58, Th: 63, IC, Pt, and Th: 60	IC and Pt :22/33Th:11/13 IC, Pt, and Th: 4/11	Ischemic: 0 Hemorrhagic: 94	Supratentorial	IC and Pt :25/30 Th:11/13 IC, Pt, and Th: 5/10	Chronic (106 days after onset)	SIAS (UE + LE) IC and Pt: 10 Th: 11 IC, Pt, and Th: 10 FIM IC and Pt: 86 Th: 87 IC, Pt, and Th: 84	1.0	T1WI T2WI	2, 4, and 6 months after onset	3 groups comparison	FIM (mobility) and the probability of ambulation without physical assistance	On admission and discharge
Moon et al., Neuroradiology, 2017^32^ (retrospective)	102	65.77 ± 13.85	60/42	Ischemic: 15 Hemorrhagic: 39	Supratentorial and infratentorial	52/41 both: 9	Subacute (≤ 90 days after onset) (mean 26.8 ± 19.2 days)	FIM: 57.58 ± 24.95	–	T1WI FLAIR	–	2 groups comparison Overlay of lesions Voxel-based lesion symptom mapping analysis Multivariate logistic regression analysis	FAC Gait speed	Baseline (≤ 90 days after onset) and after the 4-week rehabilitation
Jang et al., Ann Neurol, 2008^36^ (prospective)	25	61.6 ± 9.92	11/14	Ischemic: 25 Hemorrhagic: 0	Infratentorial	15/10	Acute to subacute (15.28 ± 6.88 days after onset,range 5–30 days)	MBC: 0.12 ± 0.33 MI: 18.96 ± 14.05	1.5	T2WI DTI	15.28 ± 6.88 days after onset (range 5–30 days)	2 groups comparison	FAC	At onset and 6 months after onset
Kim et al., NeuroRehabilitation, 2013^38^ (retrospective)	37	57.4 ± 15.2	28/9	Ischemic: 37 Hemorrhagic: 0	Supratentorial	16/21	Acute to subacute (5–30 days after onset)	MI: 7.30 ± 11.15 MBC: 1.05 ± 0.23	1.5	T2WI DTI	19.2 ± 7.5 days after onset	3 groups comparison	FAC	At onset and 6 months after onset
Soulard et al., Neurology, 2020^42^ (prospective)	29	52.14 ± 9.84	21/8	Ischemic: 29 Hemorrhagic: 0	Supratentorial	10/19	Acute (14 days after onset)	NIHSS: 13.90 ± 4.72	3	T1WI FLAIR DTI	1 month after onset	Correlation analysis	Walking score (the sum of Barthel Index (gait subscore and stairs subscore)	1, 1.5, 3, 5, 7, 13, and 25 months after onset
Baillieul et al., Hum Mov Sci, 2019^30^ (prospective)	33	63.9 ± 12.9	21/12	Ischemic: 33 Hemorrhagic: 0	Supratentorial	14/19	Acute (2.9 ± 2.7 days after onset)	NIHSS Score 0: 6 Score 1–4: 18 Score 5–15: 8 Score 16–20: 1	1.5	T1WI T2WI FLAIR	2.9 ± 2.7 days after onset	Overlay of lesions Voxel-based lesion symptom mapping analysis	Rivermead Mobility Index Gait speed Walking actibity	At 3 months after onset
Sagnier et al., Stroke, 2020^46^ (prospective)	207	66 ± 13	138/69	Ischemic: 207 Hemorrhagic: 0	Supratentorial	97/98 both: 12	Acute (within 24 to 72 h after onset)	NIHSS: median 3 (IQR: 2–6)	3	DWI FLAIR DTI	Within 24 to 72 h after onset	Multiple regression analysis Path analysis Tract-based spatial statistics analysis	Gait speed	1 year after onset
Jang et al., Int J Neurosci, 2013^37^ (not mentioned)	21	62.66 ± 8.58	6/15	Ischemic: 0 Hemorrhagic: 21	Supratentorial	12/9	Acute to subacute (16.66 ± 5.71 days after onset, range 7–30 days)	MI: 5.80 ± 7.92 MBC: 1.00 ± 0.00	1.5	T2WI DTI	16.66 ± 5.71 days after onset (range 7–30 days)	2 groups comparison Correlation analysis	FAC	At onset and 6 months after onset
Jang et al., Somatosens Mot Res, 2016^44^ (retrospective)	31	64.76 ± 10.76	12/19	Ischemic: 31 Hemorrhagic: 0	Infratentorial	20/11	Acute to subacute (12.71 ± 4.63 days after onset, range 7–28 days)	–	1.5	T2WI DTI	12.71 ± 4.63 days after onset (range 7–28 days)	Correlation analysis	FAC	At onset and 6 months after onset
Imura et al., J Phys Ther Sci, 2015^40^ (not mentioned)	25	71.5 ± 11.0	14/11	Ischemic: 16 Hemorrhagic: 9	–	–	Acute (within 10 days after onset)	–	3	DTI	Within 10 days after onset	Correlation analysis	Barthel Index (gait subscore) Functional Independence Measure (gait subscore)	1 month after onset
Burke et al., Stroke, 2014^47^ (prospective)	33	61 ± 14	–	–	–	–	Chronic (212 ± 104 days after onset)	mRS: 0.18 ± 0.46 Barthel Index: 81 ± 18	1.5	T1WI fMRI	212 ± 104 days after onset	Multivariate analysis	Gait speed Gait endurance	At baseline and 12 weeks after baseline
Lam et al., Neurorehabil Neural Repair, 2010^33^ (prospective)	52	66.8	34/18	Ischemic: 52 Hemorrhagic: 0	Supratentorial and infratentorial	20/32	Chronic (at least 6 months after onset)	NIHSS: 4.08	1.5 (n = 20) 3 (n = 32)	T1WI fMRI (n = 20)	Within 2 weeks of the start and end of the training	General linear model	Gait speed Gait endurance	Before and after training period (6 months, n = 20; 3 months, n = 32)
Loos et al., Int J Stroke, 2018^34^ (prospective)	200	66.8 ± 11.4	112/88	Ischemic: 200 Hemorrhagic: 0	–	–	–	NIHSS: 1 (range 0–7)	1.5	T1WI T2W2 FLAIR DWI	Median 7 days after onset (range: 0–142 days)	Multiple regression analysis	Timed Up and Go test Stroke impact scale (mobility domain)	3 years after onset
Smith et al., Neurorehabil Neural Repair, 2017^45^ (not mentioned)	41	Median 72 (range 43–96)	17/24	Ischemic: 35 Hemorrhagic: 6	Supratentorial and infratentorial	20/21	Acute (within 3 days after onset)	NIHSS: median 8 (range 1–21)	1.5	T1WI DWI DTI	7 to 14 days after onset	Logistic regression analysis Classification and regression tree analysis	FAC	Baseline (within 3 days after onset), 6 weeks, and 12 weeks after onset
Cho et al., Neurosci Lett, 2007^35^ (prospective)	40	53.35 ± 9.93	21/19	Ischemic: 0 Hemorrhagic: 40	Supratentorial	13/27	Acute to subacute (22.45 ± 8.04 days after onset, range 7–30 days)	MI-UE: 0.0 MI-LE: 1.0 FAC: 0	1.5	T2WI DTI	22.45 ± 8.04 days after onset (range 7–30 days)	4 groups comparison	FAC	At onset and 6 months after onset

*DTI* diffusion tensor imaging, *DWI* diffusion weighted imaging, *FAC* functional ambulation category, *FIM* functional independence measure, *FLAIR* fluid-attenuated inversion-recovery, *fMRI* functional magnetic resonance imaging, *FMA* Fugl–Meyer Assessment, *FMA-S* Fugl–Meyer Assessment sensory subscore, *IC* internal capsule, *IQR* interquartile range, *LE* lower extremity, *MI* Motricity Index, *MBC* modified Brunnstrom classification, *MRI* magnetic resonance imaging, *MRMI* modified Rivermead mobility index, *mRS* modified Rankin scale, *NIHSS* National Institutes of Health stroke scale, *Pt* putamen, *SIAS* stroke impairment assessment set, *Th* thalamus, *T1WI* T1-weighted imaging, *T2WI* T2-weighted imaging, *UE* upper extremity.

Table 2 summarizes important information from the eight structure- or function-based MRI studies, including the participants’ stroke types, MRI contents, key structure or imaging findings, and main results. All studies showed the usefulness of key structure or imaging findings in predicting gait ability. In particular, most studies indicated that damage to motor-related areas (e.g., primary motor cortex, corona radiata, internal capsule, and basal ganglia) or insula were related to poor gait recovery. Table 3 summarizes considerable information from the 11 neural tract integrity-based MRI studies, including the patients’ stroke types, MRI contents, imaging parameters, analyzed tracts, and main results. All studies included DTI-related results, and most studies showed the usefulness of a tract integrity analysis in predicting gait ability. In particular, integrity of the corticospinal tract (CST) was related to gait ability. Some studies reported the predictive value of the corticoreticular pathway (CRP).

**Table 2:** Summary of structure- or function-based MRI studies.

Study: author, journal, year	Stroke type, n	MRI contents	Key structures or imaging findings	Main results
Lee et al., Brain Behav, 2017^29^	Ischemic: 10 Hemorrhagic: 20	T1WI T2WI	Corona radiata IC Globus pallidus Putamen Cingulum Primary motor cortex Caudate nucleus	Corona radiata, internal capsule, globus pallidus, putamen, and cingulum, primary motor cortex, and caudate nucleus were related with poor gait recovery
Jones et al., Hum Brain Mapp, 2016^31^	Ischemic: 41 Hemorrhagic: 9	T1WI T2WI FLAIR	CST Putamen Insula External capsule and neighboring white matter	CST damage independently predicted response to therapy for FAC and MRMI, but not for walk speed Walk speed response to rehabilitation was affected by damage involving the putamen, insula, external capsule and neighboring white matter but not the CST
Miyai et al., Stroke, 2000^28^	Ischemic: 0 Hemorrhagic: 94	T1WI T2WI	IC Putamen Thalamus	The patients who had all 3 lesions (IC, Pt, and Th) showed greater improvement of FIM mobility scores and the probability of ambulation without physical assistance on discharge compared with the patients who had lesions in IC and Pt or Th only All patients who had all 3 lesions (IC, Pt, and Th) showed intact anterior ventral nucleus and damage in the posterior half of the internal capsule
Moon et al., Neuroradiology, 2017^32^	Ischemic: 15 Hemorrhagic: 39	T1WI FLAIR	Insula IC	Damage to the insula and internal capsule affected gait velocity change
Baillieul et al., Hum Mov Sci, 2019^30^	Ischemic: 33 Hemorrhagic: 0	T1WI T2WI FLAIR	Putamen (posterior part) IC (posterior limb) Corona radiata (anterior part)	Lower level of walking activity were related to lesions of the posterior part of putamen, posterior limb of internal capsule, and anterior part of corona radiata
Burke et al., Stroke, 2014^47^	–	T1WI fMRI	Primary sensorimotor cortex	Treatment-related gains in gait velocity were related to activation volume in ipsilesional foot primary sensorimotor cortex at baseline
Lam et al., Neurorehabil Neural Repair, 2010^33^	Ischemic: 52 Hemorrhagic: 0	T1WI fMRI (n = 20)	Subcortical lesion Left-sided lesion	10 m walk velocity improved more in the patients with subcortical rather than in the patients with cortical lesions Improvements in 6 min walk velocity were greater in the patients with left-sided lesions
Loos et al., Int J Stroke, 2018^34^	Ischemic: 200 Hemorrhagic: 0	T1WI T2W2 FLAIR DWI	Total cerebral small vessel disease burden	Total cerebral small vessel disease burden was not associated with gait impairment in all stroke patients, nor in lacunar stroke In non-lacunar stroke patients, total cerebral small vessel disease burden was associated with lower stroke impact scale (mobility domain)

*CST* corticospinal tract, *DWI* diffusion weighted imaging, *FAC* functional ambulation category, *FIM* functional independence measure, *FLAIR* fluid-attenuated inversion-recovery, *fMRI* functional magnetic resonance imaging, *IC* internal capsule, *MRI* magnetic resonance imaging, *MRMI* modified Rivermead mobility index, *Pt* putamen, *Th* thalamus, *T1WI* T1-weighted imaging, *T2WI* T2-weighted imaging.

**Table 3:** Summary of neural tract integrity-based MRI studies.

Study: author, journal, year	Stroke type, n	MRI contents	Imaging parameter	Analyzed tract	Main results
Kim et al., Neuroreport, 2018^39^	Ischemic: 40 Hemorrhagic: 8	DTI	Visual	CST	The FAC scores in the group A (CST was preserved around the lesion area) and the group B (CST was similar to group A, except that the fiber originated from cortex other than primary motor cortex) tended to be higher than that of group C (CST was interrupted or not shown)
Yeo et al., J Stroke Cerebrovasc Dis, 2020^41^	Ischemic: 0 Hemorrhagic: 9	T2WI DTI	FA MD Visual	CST CRP Medial VST Lateral VST	CST and VST did not play essential role in independent gait Intact CRP was related to the gait function
Jang et al., Ann Neurol, 2008^36^	Ischemic: 25 Hemorrhagic: 0	T2WI DTI	Visual	CST	FAC score improvement were significantly higher in DTT type A (the CST was preserved) than DTT type B (the CST was interrupted)
Kim et al., NeuroRehabilitation, 2013^38^	Ischemic: 37 Hemorrhagic: 0	T2WI DTI	Infarct volume FA ratio Visual	CST	FAC scores in group A (integrity of the CST was preserved around the infarct) were significantly higher than group B (integrity of CST was discontinuous) and group C (the upper end of the CST did not reach the infarct) There were positive correlation between FA ratio and FAC scores (r = 0.5, p = 0.002) There were negative correlation between infarct volume and FAC scores (r = − 0.361, p = 0.028)
Soulard et al., Neurology, 2020^42^	Ischemic: 29 Hemorrhagic: 0	T1WI FLAIR DTI	FA value Lesion volume	CST CRP	Walking score were correlated with lesion volume Walking score significantly correlated with FA values from ipsilesional CST, contralesional CST, ipsilesional CRP, and bilateral cerebellar peduncles Walking recovery was predicted by FA values from ipsilesional CST, ipsilesional CRP, and contralesional superior cerebellar peduncle
Sagnier et al., Stroke, 2020^46^	Ischemic: 207 Hemorrhagic: 0	DWI FLAIR DTI	Axial diffusivity FA MD Radial diffusivity	NAWM	NAWM FA was associated with gait speed (β = − 0.31, p < 0.001)
Jang et al., Int J Neurosci, 2013^37^	Ischemic: 0 Hemorrhagic: 21	T2WI DTI	FA ratio Tract length Number of fibers Visual	CST	FA ratio, fiber number ratio, and tract length ratio were positively correlated with FAC (r = 0.455, p = 0.038; r = 0.602, p = 0.004; r = 0.6, p = 0.004, respectively) FAC score in DTT type A (the CST was preserved around the hematoma) was higher than those in DTT type B (the CST was interrupted)
Jang et al., Somatosens Mot Res, 2016^44^	Ischemic: 31 Hemorrhagic: 0	T2WI DTI	FA ratio Infarct size Number of fibers Size of the CST area	CST	Fiber number ratio and CST area ration were positively correlated with FAC (r = 0.50, p = 0.004; r = 0.50, p = 0.004, respectively) There was no significant correlation between the FA ratio and FAC
Imura et al., J Phys Ther Sci, 2015^40^	Ischemic: 16 Hemorrhagic: 9	DTI	FA Number of fibers ADC	CST	There was positive correlation between the FA value of affected CST and gait parameters (gait item of Barthel Index and gait item of FIM) There was no significant correlation between other DTI parameters (ADC and number of fibers) and gait parameters (gait item of Barthel Index and gait item of FIM)
Smith et al., Neurorehabil Neural Repair, 2017^45^	Ischemic: 35 Hemorrhagic: 6	T1WI DWI DTI	FA ratio Lesion load	CST	MRI parameters were not found to have predictive value and not included in CART analysis
Cho et al., Neurosci Lett, 2007^35^	Ischemic: 0 Hemorrhagic: 40	T2WI DTI	Visual	CST	Distribution of FAC were affected by classification defined by the integrity of CST

*ADC* apparent diffusion coefficient, *CART* classification and regression tree, *CST* corticospinal tract, *CRP* corticoreticular pathway, *DTI* diffusion tensor imaging, *DWI* diffusion-weighted imaging, *FA* fractional anisotropy, *FAC* functional ambulation category, *FIM* functional independence measure, *FLAIR* fluid-attenuated inversion-recovery, *MD* mean diffusivity, *MRI* magnetic resonance imaging, *NAWM* normal-appearing white matter, *T1WI* T1-weighted imaging, *T2WI* T2-weighted imaging, *VST* vestibulospinal tract.

Table 4 summarizes the risk of bias evaluation of the included studies. In brief, two articles had five items rated as probably no (PN) or definitely no (DN). Five articles had four items rated as PN or DN. Six articles had three items rated as PN or DN. With similar rules, 1 or 5 articles had 2 or 1 items rated as PN or DN.

**Table 4:** Risk of bias evaluation of included studies in the systematic review.

	Lee et al. 2017	Kim et al. 2018	Jones et al. 2016	Yeo et al. 2020	Miyai et al. 2000	Moon et al. 2017	Jang et al. 2008	Kim et al. 2013	Soulard et al. 2020	
1. Was selection of exposed and non-exposed cohorts drawn from the same population?	PY	DY	PY	PY	DY	PY	DY	DY	PY	
2. Can we be confident in the assessment of exposure?	DY	PY	DY	PN	PN	PY	DY	PY	PN	
3. Can we be confident that the outcome of interest was not present at start of study?	DY	DY	DY	DY	DY	DY	DY	DY	DY	
4. Did the study match exposed and unexposed for all variables that are associated with the outcome of interest or did the statistical analysis adjust for these prognostic variables?	DN	PY	PY	DN	PY	PY	DY	DY	DN	
5. Can we be confident in the assessment of the presence or absence of prognostic factors?	PN	PN	DN	DN	PY	PN	PY	PY	PY	
6. Can we be confident in the assessment of outcome?	PN	PN	PN	PN	PY	PN	PY	PY	PY	
7. Was the follow up of cohorts adequate?	DY	DY	DY	DY	DY	DY	DY	DY	DY	
8. Were co-interventions similar between groups?	PY	PN	PN	PN	PY	PN	PN	PN	DN	

	Baillieul et al. 2019	Sagnier et al. 2020	Jang et al. 2013	Jang et al. 2016	Imura et al. 2015	Burke et al. 2014	Lam et al. 2010	Loos et al. 2018	Smith et al. 2017	Cho et al. 2007
1. Was selection of exposed and non-exposed cohorts drawn from the same population?	PY	PY	DY	PY	PY	PY	PY	PY	PY	DY
2. Can we be confident in the assessment of exposure?	DY	PN	PN	PY	PY	PN	PY	PY	PN	PN
3. Can we be confident that the outcome of interest was not present at start of study?	DY	DY	DY	DY	DY	DY	DY	DY	DY	DY
4. Did the study match exposed and unexposed for all variables that are associated with the outcome of interest or did the statistical analysis adjust for these prognostic variables?	DN	PY	DY	DN	DN	PY	PN	PY	PY	DY
5. Can we be confident in the assessment of the presence or absence of prognostic factors?	PN	PN	PN	DN	PY	PN	PN	PY	PY	PN
6. Can we be confident in the assessment of outcome?	PN	PN	PN	PY	PY	PN	PN	PY	PY	PN
7. Was the follow up of cohorts adequate?	DY	DY	DY	DY	DY	PN	DY	PY	DY	DY
8. Were co-interventions similar between groups?	PN	PN	PN	PN	PN	DN	DN	PN	PY	PN

*DY* definitely yes (low risk of bias), *PY* probably yes, *PN* probably no, *DN* definitely no (high risk of bias), *N/A:* not applicable.

## Discussion

The present systematic review aimed to evaluate the usefulness of MRI in predicting the gait ability of stroke patients. Out of the 19 studies that met our criteria, eight were classified as structure- or function-based MRI studies and 11 as neural tract integrity-based MRI studies. All included studies had some concerns, at least one, based on the Cochrane risk of bias instrument.

The eight structure- or function-based MRI studies showed that MRIs are useful in predicting gait ability. Overall, most studies revealed that the patients who had damage to their motor-related structures—that is to say, component structures of the CST (primary motor cortex, corona radiata, and internal capsule) or basal ganglia (caudate nucleus, putamen, and globus pallidus)—showed poor gait recovery^28–30^. Interestingly, Jones et al.^31^ reported that CST damage independently predicted the response to therapy for general mobility ability, defined using the functional ambulation category and the modified Rivermead mobility index, but not walk speed. Alternatively, they showed that the walk speed response to rehabilitation was affected by damage involving the putamen, insula, external capsule, and neighboring white matter, but not the CST. Moon et al.^32^ investigated the predictors of gait velocity change and the association between a lesion location and a change in the gait function. As a result, they concluded that damage to the insula, in addition to the internal capsule, affected the gait velocity change after rehabilitation. Moreover, it has already been suggested that improvements in 6-min walk velocity were greater in those patients with left-sided lesions^33^. In short, from the perspective of structure- or function-based MRI studies, damage to CST-related structures was associated with fundamental gait ability, defined using a functional ambulation category or modified Rivermead mobility index^31^, while improvement of more applicative gait ability (e.g., gait velocity) seemed to be present in those patients with an intact basal ganglia, insula, or external capsule and left-sided lesions^31,33^. In addition, it has already been suggested that the total cerebral small vessel disease burden in non-lacunar stroke patients is associated with gait impairment^34^, indicating that such findings should be carefully observed adding to damage to motor-related structures.

Out of the 11 neural tract integrity-based MRI studies, 10 showed usability of MRIs in predicting gait ability. Several previous studies suggested that patients whose CST was visually preserved showed better walking recovery compared to those whose CST was interrupted or not shown, regardless of differences in brain infarctions or hemorrhaging^35–39^. Additionally, a significant correlation was observed between the fractional anisotropy value of CST and gait recovery^37,38,40^. Interestingly, in contrast, Yeo et al.^41^ showed that neither the CST nor vestibulospinal tract played an important role in independent gait, but an intact CRP was related to gait function in 9 patients with pontine hemorrhage. They demonstrated the important relationship exists between the CRP, not the CST, and gait ability although the lack of relationship between the CST and walking ability might be affected by the limited sample size. Soulard et al.^42^ also suggested the importance of CRP for gait prediction. The corticoreticulospinal tract, which consists of the CRP and the reticulospinal tract, is a well-known neural network for walking and proximal muscle regulation^43^. No consensus was obtained regarding the predictive value of fiber number-related parameters^37,40,44^. The findings from the neural tract integrity-based MRI studies were summarized that the CST integrity evaluated by DTI were basically thought as a useful predictor. Remarkably, even those patients who was not described the CST by DTI, clinician need to bear in mind that there might be still possibility for regaining walking ability if the CRP was not destruction. Smith et al.^45^ performed a classification and regression tree analysis with various variables such as physical functions, neurophysiological findings using transcranial magnetic stimulation, and MRI information to identify the factors that predict time to independent walk. As a result, TMS and MRI measures did not have predictive value.

Regarding the risk of bias evaluation of included studies, thirteen of nineteen articles were rated as PN or DN in more than three items. In particular, the items that evaluate the assessment of the presence or absence of prognostic factors and the concerning of co-interventions between groups were rated as PN or DN in many articles. Moreover, none of the included articles investigated the additional value into other predictors or the competitive advantage of the use of MRIs in predicting gait ability of stroke patients. With these consideration in mind, it is expected that further studies will be performed to consolidate strong evidence.

This study has certain limitations. First, we might have missed some relevant studies because our search strategy consisted of selected words and databases. Second, we only included studies that were published in the English language; therefore, we have to consider relevant language biases and the limited generalizability of the present results. Third, we could not apply a quantitative analysis in this review, because the included studies were heterogeneous. Fourth, most included studies, even those showing usefulness of MRIs for gait prediction, did not investigate the additional value into other basic predictors or the competitive advantage throughout comparison with other clinical basic variables. Despite these limitations, to our knowledge, this is the first report to consolidate evidence regarding the usefulness of MRIs in predicting the gait ability of stroke patients.

In conclusion, the present systematic review suggests that MRIs are useful in predicting the gait ability of stroke patients. We were able to suggest important findings for predicting gait ability from an MRI. However, we cannot make definitive conclusions regarding the predictive value and effects of gait prediction using MRI findings, due to the lack of quantitative evaluations. Therefore, more high-quality studies are needed related to gait prediction using MRIs, including verification of their predictive accuracy.

## Supplementary Information

Supplementary Information 1.

Supplementary Information 2.
