# Supplementary material for: A systematic review of the usefulness of magnetic resonance imaging in predicting the gait ability of stroke patients
Source: Sci Rep. 2021 Jul 12;11:14338. doi: 10.1038/s41598-021-93717-4 (PMC8275756; doi:10.1038/s41598-021-93717-4)
Supplement: Supplementary file 1 — Supplementary Information 1. [file 41598_2021_93717_MOESM1_ESM.pdf]

Supplementary File 1. Search strategy

A systematic review of the usefulness of magnetic resonance imaging in predicting the gait ability of stroke patients

Takeshi Imura*, PhD[1], Tsubasa Mitsutake, PhD[2], Yuji Iwamoto, M.S. [3], Ryo Tanaka, PhD[3]

Supplementary File 1. Search strategy

		Search number	Query
NOT search		#15	Search (#4 and #9 and #13) NOT ("animals"[MeSH Terms:noexp] NOT "humans"[MeSH Terms])NOT "healthy"[Title/Abstract]) NOT "child"[MeSH Terms]) NOT "child"[Title/Abstract]
P and E and O		#14	Search (#4 and #9 and #13)
O	Gait performance	#13	Search (#10 or #11 or #12)
		#12	((Walking[MeSH Terms]) OR (Walking[Title/Abstract])) OR (Ambulation[Title/Abstract])
		#11	((Gait[MeSH Terms]) OR (Gait[Title/Abstract])) OR (Gaits[Title/Abstract])
		#10	((((((((Clinical Decision Rules[MeSH Terms]) OR (Clinical Decision Rules[Title/Abstract])) OR (Clinical Decision Rule[Title/Abstract])) OR (Decision Rule, Clinical[Title/Abstract])) OR (Clinical Prediction Rule[Title/Abstract])) OR (Prediction Rule, Clinical[Title/Abstract])) OR (Rule, Clinical Prediction[Title/Abstract])) OR (Prediction[Title/Abstract])) OR (Clinical prediction[Title/Abstract])
E	Magnetic resonance neuroimaging	#9	Search (#5 or #6 or #7 or #8)
		#8	((((((((Diffusion Tensor Imaging[MeSH Terms]) OR (Diffusion Tensor Imaging[Title/Abstract])) OR (Imaging, Diffusion Tensor[Title/Abstract])) OR (Diffusion Tensor Magnetic Resonance Imaging[Title/Abstract])) OR (Diffusion Tensor MRI[Title/Abstract])) OR (MRI, Diffusion Tensor[Title/Abstract])) OR (DTI MRI[Title/Abstract])) OR (Diffusion Tractography[Title/Abstract])) OR (Tractography, Diffusion[Title/Abstract])
		#7	(((((Diffusion Magnetic Resonance Imaging[MeSH Terms]) OR (Diffusion Magnetic Resonance Imaging[Title/Abstract])) OR (Magnetic Resonance Imaging, Diffusion[Title/Abstract])) OR (Diffusion MRI[Title/Abstract])) OR (Diffusion Weighted MRI[Title/Abstract])) OR (MRI, Diffusion Weighted[Title/Abstract])
		#6	(((((((((((((((Magnetic Resonance Imaging[MeSH Terms]) OR (Magnetic Resonance Imaging[Title/Abstract])) OR (Imaging, Magnetic Resonance[Title/Abstract])) OR (NMR Imaging[Title/Abstract])) OR (Imaging, NMR[Title/Abstract])) OR (MR Tomography[Title/Abstract])) OR (Steady State Free Precession MRI[Title/Abstract])) OR (Zeugmatography[Title/Abstract])) OR (Chemical Shift Imaging[Title/Abstract])) OR (Proton Spin Tomography[Title/Abstract])) OR (Magnetization Transfer Contrast Imaging[Title/Abstract])) OR (MRI Scan[Title/Abstract])) OR (fMRI[Title/Abstract])) OR (Functional MRI[Title/Abstract])) OR (Functional Magnetic Resonance Imaging[Title/Abstract])) OR (Spin Echo Imaging[Title/Abstract])

		#5	(((Neuroimaging[MeSH Terms]) OR (Neuroimaging[Title/Abstract])) OR (Brain Imaging[Title/Abstract])) OR (Imaging, Brain[Title/Abstract])
P	Stroke	#4	Search (#1 or #2 or #3)
		#3	((Intracranial Hemorrhage[MeSH Terms]) OR (Intracranial Hemorrhage[Title/Abstract])) OR (Posterior Fossa Hemorrhage[Title/Abstract])
		#2	(((((((Brain Infarction[MeSH Terms]) OR (Brain Infarction[Title/Abstract])) OR (Anterior Circulation Brain Infarction[Title/Abstract])) OR (Venous Infarction[Title/Abstract])) OR (Brain Venous Infarction[Title/Abstract])) OR (Venous Brain Infarction[Title/Abstract])) OR (Anterior Cerebral Circulation Infarction[Title/Abstract])) OR (Posterior Circulation Brain Infarction[Title/Abstract])
		#1	(((((((((((Stroke[MeSH Terms]) OR (Stroke[Title/Abstract])) OR (Cerebrovascular Accident[Title/Abstract])) OR (CVA[Title/Abstract])) OR (Cerebrovascular Apoplexy[Title/Abstract])) OR (Vascular Accident[Title/Abstract])) OR (Brain Vascular Accident[Title/Abstract])) OR (Cerebrovascular Stroke[Title/Abstract])) OR (Apoplexy[Title/Abstract])) OR (Cerebral Stroke[Title/Abstract])) OR (Acute Stroke[Title/Abstract])) OR (Acute Cerebrovascular Accident[Title/Abstract])
