# Supplementary material for: A systematic review of the usefulness of magnetic resonance imaging in predicting the gait ability of stroke patients
Source: Sci Rep. 2021 Jul 12;11:14338. doi: 10.1038/s41598-021-93717-4 (PMC8275756; doi:10.1038/s41598-021-93717-4)
Supplement: Supplementary file 2 — Supplementary Information 2. [file 41598_2021_93717_MOESM2_ESM.pdf]

Supplementary File 2. Predetermined criteria for risk of bias evaluation

A systematic review of the usefulness of magnetic resonance imaging in predicting the gait ability of stroke patients

Takeshi Imura*, PhD[1], Tsubasa Mitsutake, PhD[2], Yuji Iwamoto, M.S. [3], Ryo Tanaka, PhD[3]

Supplementary File 2. Predetermined criteria for risk of bias evaluation

	DY (low risk of bias)	PY	PN	DN (high risk of bias)
1. Was selection of exposed and non-exposed cohorts drawn from the same population?	Cohort study divided by exposed factor	Cohort study without grouping	Case-control study	NA
2. Can we be confident in the assessment of exposure?	Meet all criteria<br>1. The reliability has been established<br>2. Evaluator was blinded to other data<br>3. One skilled evaluator or more than 2 evaluators	Meet 2 criteria in the left column	Meet 1 criteria in the left column	Not mentioned
3. Can we be confident that the outcome of interest was not present at start of study?	All included studies were defined as DY because of our selection criterion (4)	—	—	—
4. Did the study match exposed and unexposed for all variables that are associated with the outcome of interest or did the statistical analysis adjust for these prognostic variables?	Meet the all criteria<br>1. Group comparison<br>2. The similarity of all variables except the exposed factor between groups was confirmed	Meet any criteria<br>1. Group comparison, but did not meet the criteria in the left column<br>2. ANCOVA, multiple regression analysis, logistic regression analysis with all plausible variables, or subgroup analysis	Meet any criteria<br>1. ANCOVA, multiple regression analysis, logistic regression analysis with some plausible variables, or subgroup analysis<br>2. The variables between groups showed similarity as haphazard, without analysis	Not confirmed
5. Can we be confident in the assessment of the presence or absence of prognostic factors?	Evaluated prognostic factors (at least 1 factor) meet all criteria<br>1. The reliability has been established<br>2. Evaluator was blinded to other data<br>3. One skilled evaluator or more than 2 evaluators	Meet 2 criteria in the left column	Meet 1 criteria in the left column	Not mentioned
6. Can we be confident in the assessment of outcome?	Evaluated outcomes (at least 1 outcome) meet all criteria<br>1. The reliability has been established<br>2. Evaluator was blinded to other data<br>3. One skilled evaluator or more than 2 evaluators	Meet 2 criteria in the left column	Meet 1 criteria in the left column	Not mentioned
7. Was the follow up of cohorts adequate?	More than 85% of participants were followed up	More than 75% of participants were followed up	More than 65% of participants were followed up	More than 55% of participants were followed up
8. Were co-interventions similar between groups?	The content of co-interventions between participants was strictly equal.	The content of co-interventions is mentioned (single-center study)	One of the following<br>1. There was no mention about the content of co-interventions.<br>2. The content of co-interventions is mentioned (multi-center study)	There was no equalization of co-interventions

ANCOVA, analysis of covariance; DN, definitely no; DY, definitely yes; NA, not applicable; PN, probably no; PY, probably yes
